# The Birmingham Scheme of Classified Institutions

**Published:** 1914-11-21

**Authors:** 


### THE BIRMINGHAM SCHEME OF CLASSIFIED INSTITUTIONS.

### What it Does, and Where it Fails.

A special classification committee of the Birmingham Guardians has recently presented an important report on the classification of the inmates in the institutions of the Board. The report deals with the inmates of the workhouses and infirmaries, and is based mainly on recommendations made by Dr. Ellis, the chief medical officer.

### Dr. Ellis's Report.

The details of the scheme as regards the allocation of the classes to the various institutions are of merely local interest, but the principles upon which the proposed classification is founded may be studied with advantage by everyone concerned with Poor-Law administration. Dr. Ellis's report shows that he has devoted a very great deal of care and thought to the subject, and the masterly manner in which he has solved the problem as it presents itself at Birmingham, within the limitations imposed upon him by the conditions obtaining there, is worthy of the highest praise. The scheme he has worked out is based mainly upon the physical condition of the inmates, but, in the case of the workhouses, he has provided for separate classes for the meritorious and the vicious; the test for merit being applied in the main, very rightly, to the conduct of the inmate whilst under the care of the Guardians. He first divides the inmates into the following groups: (1) Children, (2) the able-bodied adults, (3) those capable of doing a certain amount of work but physically or mentally below the average standard demanded by an employer of labour, (4) those temporarily disabled from work by trivial ailments, (5) the infirm who are able to be up all day but incapable of work on account of their infirmity, (6) chronic cases wholly or partially bedridden, (7) the hospital group, (8) maternity cases and suckling mothers, (9) epileptics and feeble-minded, and (10) the convalescents. The mere enumeration of these groups shows how very varied are the classes which come under the survey of the Poor-Law administrator. Dr. Ellis makes several interesting suggestions as regards the children.

### The Provision for Training.

He proposes that all cripple children capable of being benefited by remedial physical exercises should be sent to Erdington so that they may attend the Fentham Road Schools, where such exercises are properly carried out by trained teachers, and that an open-air school should be provided for delicate children. Special provision is to be made for pre-maternity cases and for suckling mothers. These are to be classified according to merit, but not on a simple basis of married and unmarried. This method of division will commend itself to all experienced administrators. Cases when in labour are to be sent to two of the infirmaries where maternity blocks are provided. Special provision is to be made for suckling mothers who will agree to stay for about twelve months. They are to be trained to laundry work in the institution, and are to receive instruction in the care of their infants and in making their clothes. Dr. Ellis hopes that the training thus given them will enable them on their discharge to earn their own living, and will awaken in them a fuller sense of the responsibility of motherhood. He lays stress upon the paramount importance to the child of affording the mother facilities for nursing it, and the moral effect this natural association with the child would have upon the mother.

The Birmingham Guardians have had the wisdom to provide an excellent colony for epileptics and feeble-minded at Monyhull. It is proposed that all cases of this class shall be admitted primarily to the Dudley Road Infirmary, whence the bulk will be drafted to Monyhull. Those unsuited for a colony will be sent to Erdington Workhouse or to the asylum as may be necessary.

### Relief for the Infirmaries.

One of the main objects of the classification scheme is to relieve the pressure upon the infirmaries of the Union. Dr. Ellis proposes to do this by restricting the use of the infirmaries to those suffering from acute illness or from conditions capable of being benefited by active medical or surgical treatment. Chronic cases, including ulcered legs which do not respond to treatment and nervous and other cases unlikely to improve or not requiring skilled nursing, would be transferred to the workhouses. That, whatever the cause may be, the average stay of patients in the infirmaries is unduly prolonged is shown by the fact that in 1913, with an average number of occupied beds of 1,022 at Dudley Road Infirmary and 397 at the Selly Oak Infirmary, only 4,756 patients were admitted to the former and 1,001 to the latter institution. Whether this long average duration of treatment is entirely due to the chronic nature of the cases admitted, it is impossible to say upon the facts presented. We suspect, however, that it is partly due to the great defect of Poor-Law administration as regards the sick—insufficient staffing and the lack of properly equipped special departments. We feel that many of the cases which Dr. Ellis labels chronic and proposes to relegate to the workhouses might under energetic and expert treatment be saved from drifting into a condition of permanent disability.

### The Price for having no Specialist Staff.

Let us take as one example the chronic nervous cases. In doing so let us make it clear at the outset that we do not know how far our criticisms may apply to the institutions of the Birmingham Guardians. They may be exceptionally well provided for in this respect, but we do say this, that the great majority of Poor-Law infirmaries have no modern neurological department. They have no neurologist, visiting or resident, on the staff.

They have no department in which massage and treatment by electricity and systematised exercises can be carried out. Under these conditions who shall say how many cases exist in them of unrecognised spinal and cerebral tumours or of cervical ribs, which might be cured by operation, but are classed, as the result of the inadequate examination an overworked staff can give them, as hopeless and progressive cases of nervous disorder? How many infirmaries are there which are able even to give to their tabetic patients the advantages to be derived from the simple exercises devised by Frenkel? How many are there that attempt to benefit their cases of spastic contraction or of tabes by the operation of posterior nerve-root section? We know the answer to these questions. It would be very, very few indeed. And this answer is no discredit to the present Poor-Law infirmary staffs. It simply means that they are not properly manned and equipped for their work. We could ask similar questions as regards other of the chronic cases which fill our infirmaries, only to receive the same replies.

The Infirmary Problem not Solved.

Classification on the Birmingham model would have this effect. It would release many beds for acute cases and would enable the staff to concentrate their attention upon them, but it would not solve the problem of the Poor-Law infirmary. That problem can be solved only by an increased and specialised staff. A medical superintendent with two or three junior medical officers cannot deal properly nowadays with the variety of cases which crowd into a Poor-Law infirmary of 500 beds. To divide them simply, as Birmingham proposes, into acute and chronic cases—in other words, into those which can be treated by a general surgeon, assisted by general practitioners, and those which cannot—may be of advantage from an administrative point of view, but we know that more can be done and ought to be done. For the sake of economy we must have classification so that we may not waste expensive accommodation upon senile cases and others for which at present medical science can afford no amelioration. But, first, we must be sure that our infirmaries are so staffed that no patient is classed as incurable until his case has been adequately investigated by all the special methods we now have at our command. Poor-Law Guardians and the Local Government Board have never apparently realised it, but the first requirement of efficient medical treatment is not the building, nor the equipment, nor methods of classification, but the expert knowledge of the staff using them. A trained and experienced surgeon can do much with a knife and a needle and thread; an untrained man can do nothing with the most expensively equipped operating theatre and cabinet of instruments.

